# Analysis of Chemical Composition in Three Types of *Fritillaria* Using UPLC–Q‐TOF‐MS/MS Technology

**DOI:** 10.1155/ianc/9972411

**Published:** 2026-04-29

**Authors:** Shuang Ji, Xufeng Mao, Desheng Qi, Shaoxiong Zhang, Zhi Chen, Feng Qiao, Zhiqiang Dong, Shaobo Du, Huichun Xie

**Affiliations:** ^1^ School of Geographic Sciences, Qinghai Normal University, Xining, 810008, China, qhnu.edu.cn; ^2^ Key Laboratory of Medicinal Animal and Plant Resources on the Qinghai–Tibet Plateau, Xining, 810008, China; ^3^ National Positioning Observation and Research Station for Forest Ecosystem on the South Slope of the Qilian Mountains in Qinghai, Huzhu, 810500, China; ^4^ School of Life Sciences, Qinghai Normal University, Xining, 810008, China, qhnu.edu.cn

## Abstract

Understanding the chemical composition of plants is essential for elucidating their evolutionary adaptations and evaluating the pharmaceutical potential of their primary and secondary metabolites. In this study, we compared the primary and secondary metabolites of three closely related *Fritillaria* species from the Qinghai–Tibetan Plateau using ultra‐performance liquid chromatography coupled with quadrupole time‐of‐flight mass spectrometry (UPLC–Q‐TOF‐MS/MS). Chemical constituents were qualitatively identified based on accurate mass measurements combined with MS^1^ and MS^2^ fragmentation information. A total of 72 compounds were identified across the three *Fritillaria* species. All species exhibited rich chemical diversity and demonstrated considerable medicinal potential. The differential metabolites included several bioactive compounds, such as peimine and verticine 3‐glucoside, among others. In addition, *F. thunbergii* showed unique alkaloids, including zhebeinine, distinguishing it from the other two species. These findings provide a valuable basis for understanding differences in pharmacological metabolism among the three *Fritillaria* species and highlight newly reported constituents that expand the known chemical diversity of the genus, offering potential implications for species discrimination, quality evaluation, and rational resource utilization.

## 1. Introduction

Understanding the detailed chemical composition of plants is essential for understanding their evolutionary adaptations, including the composition of primary and secondary metabolites [1]. While plant primary metabolites are essential for growth and reproduction, secondary metabolites (natural products or specialized metabolites) play an important role in plant defense against various constraints, including harsh climatic conditions and plant–herbivore interactions. Secondary metabolites are often restricted to certain taxa (hence the term “specialized metabolites”) and help plants survive harsh environmental conditions such as heavy grazing or low temperatures in high mountain regions.

*Fritillaria*, native to the high mountains of central and southeastern China [2], is considered an important protected wild medicinal plant. Among different species of *Fritillaria*, *F. cirrhosa*, *F. unibracteata*, and *F. thunbergii* are mainly distributed in the provinces of Qinghai, Gansu, Tibet, and Sichuan [2], and their bulbs are widely used in traditional folk medicine to treat typhoid fever, dysuria, mental disorders, cough, hernia, and throat obstruction [3–5]. Modern pharmacological studies have shown that these *Fritillaria* species exhibit antitussive, expectorant, antiasthmatic, anti‐inflammatory, and antitumor activities [6–10].

Currently, *Fritillaria* species can be distinguished based on morphological characteristics such as flower color and leaf patterns. However, the medicinal part of *Fritillaria* is the bulb, and it is difficult to distinguish dried bulbs sold in the market based on their external features, leading to mixed use and misuse of medicinal materials derived from different *Fritillaria* species [11]. Previous research indicates that *F. cirrhosa*, *F. unibracteata*, and *F. thunbergii* differ in their pharmacodynamic properties [12]. *F. cirrhosa* is mainly used for moistening the lungs and relieving cough and asthma caused by lung dryness. In contrast, *F. thunbergii* is used for clearing heat, resolving phlegm, and detoxification, especially for lung conditions associated with heat and phlegm–heat congestion. Notably, *F. unibracteata*, a subset within *F. cirrhosa*, is considered a valuable source with stronger therapeutic effects in relieving cough, eliminating phlegm, and alleviating asthma than *F. cirrhosa* and is regarded as a representative species of the *F. cirrhosa* group. However, the reasons underlying these pharmacodynamic differences among the three *Fritillaria* species remain unclear. To ensure the sustainable development and utilization of *Fritillaria* species while safeguarding germplasm resources and standardizing the cultivation of authentic medicinal materials, systematic and comprehensive scientific evaluation is needed to clarify the pharmacological material basis of the genus *Fritillaria*.

The chemical constituents of *Fritillaria* are complex and diverse, including alkaloids, nucleosides, fatty acids, carbohydrates, and other compounds [3, 13–15]. Currently, research on the chemical constituents of *Fritillaria* mainly focuses on determining chemical fingerprints [16], analyzing components of compound prescriptions [17], and performing quantitative and qualitative analysis of characteristic constituents [18, 19]. However, a comprehensive qualitative analysis of the chemical compounds in *Fritillaria* is still lacking.

Traditional Chinese medicine (TCM), characterized by complex compositions and diverse therapeutic targets, poses challenges in identifying its therapeutic material basis and ensuring quality control using conventional DNA barcoding methods [20]. Understanding the chemical composition of TCM is therefore crucial for elucidating its therapeutic effects, mechanisms, and clinical efficacy. Hence, there is a need for precise, efficient, and rapid analytical methods. In recent years, UPLC–Q‐TOF‐MS has emerged as a powerful tool for analyzing the chemical constituents of Chinese herbal medicines. This widely used technology facilitates quality control, structural identification, and modernization of herbal medicine analysis [21]. For example, Lou et al. [22] used UPLC–Q‐TOF‐MS to analyze characteristic metabolites in two *Dendrobium* species, identifying 58 chemical components and significant differences between them. Similarly, Han et al. [23] comprehensively analyzed the chemical components of Huangqi Guizhi Wuwu Tang, providing marker compounds for establishing quality standards.

In this study, to further elucidate the pharmacologically relevant compounds in the three *Fritillaria* species, we used UPLC–Q‐TOF‐MS/MS technology to comprehensively analyze their chemical constituents combined with metabolomic analysis, providing new insights into species‐specific chemical characteristics while expanding the known chemical diversity of the genus through newly reported constituents, with potential implications for pharmacological differentiation, quality evaluation, and sustainable resource utilization of *Fritillaria*.

## 2. Materials and Methods

### 2.1. Sample Collection

Three species of *Fritillaria* were collected for analysis in this study. Two species, *F. unibracteata* (AZ) and *F. cirrhosa* (CB), were collected in the field from June to August 2020 at 12 different localities within Qinghai Province, China (Table 1). These localities represent typical habitats of *Fritillaria*, specifically subalpine meadows (3000–4500 m) on the Qinghai–Tibetan Plateau, which experience a mean annual temperature of −10°C to 4°C and a mean annual precipitation of 473–550 mm.

**TABLE 1:** Collection information of *F. unibracteata* and *F. cirrhosa*.

No.	Locality	Altitude/m	Longitude (*E*)	Latitude (*N*)
AZ‐1	Guoluo Prefecture^a^	3505 m	10042′13″	3238′65″
AZ‐2	Guoluo Prefecture^b^	3669 m	10038′34″	3313′19″
AZ‐3	Yushu Prefecture^a^	3762 m	9688′09″	3232′91″
AZ‐4	Yushu Prefecture^b^	3880 m	9651′09″	3209′55″
AZ‐5	Guoluo Prefecture^c^	4266 m	10052′36″	3317′03″
AZ‐6	Huangnan Prefecture^a^	3042 m	10155′52″	3504′56″
CB‐1	Yushu Prefecture^c^	4230 m	9225′44″	3319′23″
CB‐2	Yushu Prefecture^d^	4120 m	9727′31″	3306′55″
CB‐3	Yushu Prefecture^e^	4357 m	9722′46″	3326′31″
CB‐4	Guoluo Prefecture^d^	4365 m	10051′85″	3317′82″
CB‐5	Haibei Prefecture^a^	3135 m	9944′08″	3820′07″
CB‐6	Haibei Prefecture^b^	3209 m	9952′04″	3816′49″

*Note:* a∼e represent collection locations in different areas within the same autonomous prefecture.

The third species, *F. thunbergii* (ZB), was purchased from Shenzhen Heshun Bencao Pharmaceutical Co., Ltd. (China; batch numbers: C18034301–C18034306) and originated from Pan’an County, Zhejiang Province. For each species, a total of six samples were collected for subsequent analyses. The identities of *F. unibracteata* and *F. cirrhosa* were confirmed based on botanical morphological characteristics using a stereomicroscope (SZ61, Olympus, Japan). The identity of *F. thunbergii* was verified based on supplier documentation and morphological examination of the dried bulbs.

### 2.2. Preparation of Test and Reference Samples

The belowground bulbs of each plant individual were naturally dried, ground into powder using a grinder (FW100, Taisite Instrument Co., China) and stored at −20°C. From each sample, 0.8 g of powder was immersed in 3 mL of methanol (HPLC grade, Merck, Germany) and ultrasonicated for 2 h in an ultrasonic bath (KQ‐500E, Kunshan Ultrasonic Instruments Co., China), followed by incubation at 4°C for 12 h. The samples were then placed in a 50°C water bath (HH‐4, Shanghai Lichen Instrument Co., China) for 30 min before being ultrasonicated again for 1 h. After shaking and allowing the samples to stand for 5 min, 300 μL of the supernatant was mixed with 900 μL of 75% acetonitrile (HPLC grade, Thermo Fisher Scientific, USA; v/v = 3:1) and centrifuged for 10 min at 12,000 rpm and 10°C using a refrigerated centrifuge (5430R, Eppendorf, Germany). Three subsamples of each supernatant (> 600 μL each) were transferred into 2 mL Eppendorf tubes and labeled as AZ01–AZ06 (*n* = 6), CB01–CB06 (*n* = 6), and ZB01–ZB06 (*n* = 6) for subsequent analyses. For the quality control sample, 60 μL of each solution was collected, pooled thoroughly, and labeled as BM‐QC.

Following the guidelines of the Chinese Pharmacopoeia (2020), reference substances including imisine (PCS‐201112), edpetiline (PCS‐20824), sipeimine (PCS‐200523), peimine (PCS‐200728), and peiminine (PCS‐200413) were selected based on their active ingredients. All reference substances were purchased from Chengdu Zhibiao Huapu Biotechnology Co., Ltd., (China), and their purity was verified by HPLC. Each reference substance was dissolved in methanol (HPLC grade, Merck, Germany) to prepare a stock solution at a concentration of 1 mg mL^−1^. Then, 25 μL of each stock solution was diluted with 1000 μL of 75% acetonitrile (HPLC grade, Thermo Fisher Scientific, USA). After centrifugation for 10 min at 12,000 rpm and 10°C (5430R, Eppendorf, Germany), the supernatant (> 600 μL) of each reference solution was transferred into 2 mL Eppendorf tubes and labeled separately for subsequent analyses.

### 2.3. UPLC–Q‐TOF‐MS Analysis

UPLC–Q‐TOF‐MS analyses were performed using a Xevo G2‐XS QToF mass spectrometer equipped with a Z‐Spray electrospray ionization (ESI) source and MassLynx 4.1 software (Waters Corp., Milford, MA, USA). Chromatographic separation was achieved on an ACQUITY UPLC HSS T3 C18 column (2.1 × 100 mm, 1.8 μm; Waters Corp., Milford, MA, USA). The mobile phases consisted of 0.1% formic acid in water (A) and 0.01% formic acid in acetonitrile (B), delivered at a flow rate of 0.3 mL min^−1^. The column temperature was maintained at 40°C, and the autosampler was set at 10°C. The injection volume was 1 μL for all samples.

The elution gradient was programmed as follows: 0–1 min, 1%–20% B; 1–7 min, 20%–35% B; 7–9 min, 35%–45% B; 9–10 min, 45%–50% B; 10–11 min, 50%–60% B; 11–17 min, 60%–65% B; 17–18 min, 65%–99% B; 18–21 min, 99%–1% B; and 22–23 min, 1%–100% B. Three technical replicates were injected for each sample, including a blank control consisting of 75% acetonitrile. The injection sequence was randomized to minimize potential instrumental drift. Weak and strong needle‐wash solvents consisted of 90:10 and 10:90 (v/v) mixtures of water and acetonitrile, respectively.

The ESI source operated in both positive‐ion (+) and negative‐ion (−) modes over a mass range of m/z 50–1200 with a scan time of 0.2 s and a total acquisition time of 20 min. A mixture of leucine–enkephalin (200 pg μL^−1^, Waters Corp., USA) and sodium formate (0.5 mM, Sigma–Aldrich, USA) was used as the reference solution, generating reference ions at m/z 556.2771 (ESI+) and m/z 554.2615 (ESI–) at a constant flow rate of 20 μL min^−1^.

Low‐energy collision voltage was set to 6 V, and high‐energy collision voltage ranged from 20 to 60 V for data acquisition. Additional ESI parameters were as follows: capillary voltage (positive mode), 3.0 kV; cone voltage, 65 V; source temperature, 100°C; desolvation temperature, 450°C; cone gas (*N*_2_) flow rate, 50 L h^−1^; and desolvation gas (*N*_2_) flow rate, 800 L h^−1^.

### 2.4. Data Processing

Data processing was performed based on measured accurate mass values, retention times (tRs), MS^1^ and MS^2^ fragmentation patterns, as well as previously reported MS information from the literature [23]. Additional annotation resources included ChemSpider, PubChem, MassBank, and TCM databases (Chinese Medicine Database, UNIFI 1.7; Waters Corp., Milford, MA, USA). Compound discrimination and identification were conducted by integrating database searches with published literature.

Raw data were converted and preprocessed using Progenesis QI for LC/MS (Nonlinear Dynamics, Waters Corp., USA), including peak extraction, alignment, identification, and normalization. Multivariate statistical analyses, including principal component analysis (PCA) and orthogonal partial least squares discriminant analysis (OPLS‐DA), were performed using EZinfo software (Version 3.0, Waters Corp., USA). PCA was used to visualize overall variability and group clustering among the samples based on their complete metabolite profiles. In contrast, OPLS‐DA was applied to classify samples according to the three *Fritillaria* species and to identify discriminant metabolites contributing to group separation.

Differential metabolites were screened by integrating the S‐plot derived from the OPLS‐DA model and the variable importance in projection (VIP) values. Metabolites with VIP > 1 were considered influential to the model, with higher VIP values indicating greater contribution. To obtain metabolites showing more pronounced differences and improved detectability, relatively stringent screening criteria were adopted (VIP > 5 in positive‐ion mode and VIP > 4 in negative‐ion mode), enabling the selection of metabolites with stronger discriminatory power while reducing potential noise in multivariate modeling. VIP‐based variable selection in OPLS‐DA is widely used in chemometrics and metabolomics to identify variables contributing most to class separation; therefore, stricter VIP cutoffs were applied here to prioritize robust discriminatory features [24, 25].

## 3. Results and Discussion

### 3.1. QC and TIC in Positive‐ and Negative‐Ion Mode

The total ion chromatograms (TICs) of the QC samples were visually compared to assess potential analytical variability arising from instrument instability or large sample batches. The QC chromatograms showed highly consistent response intensities and tRs across all peaks, indicating good system stability and confirming both the reliability of the experimental procedure and the proper performance of the UPLC–Q‐TOF‐MS system.

### 3.2. Analysis of Chemical Constituents of Three Species of *Fritillaria*

TICs were generated for each of the three species (*F. unibracteata*, *F. cirrhosa*, and *F. thunbergii*). The results revealed clear differences among the species in both the composition and relative abundance of major chemical constituents. Similar species‐specific variations in chemical profiles have been previously reported. For example, a metabolomic study of *F. cirrhosae* Bulbus from multiple sources showed statistically distinct metabolite signatures across species using UPLC‐QTOF‐MS/MS and multivariate analysis [26]. To further characterize these interspecies differences, multivariate statistical analyses (e.g., OPLS‐DA and PCA) were applied, following the approach used in other *Fritillaria* quality‐control studies [27]. Based on visual inspection of the TICs—considering peak intensity, resolution, and overall ion signal quality—chromatograms from samples AZ‐3, CB‐5, and ZB‐1 were selected as representative profiles, as they exhibited signal characteristics consistent with the overall dataset. These representative samples were then imported into the UNIFI platform for comprehensive characterization of the chemical constituents of the three *Fritillaria* species (Figure 1).

FIGURE 1TIC of three types of *Fritillaria* and control samples in positive‐ion (a) and negative‐ion (b) modes.

**Figure:**
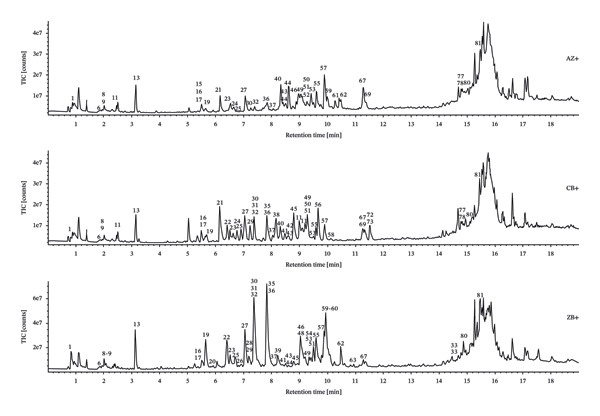
(a)

**Figure:**
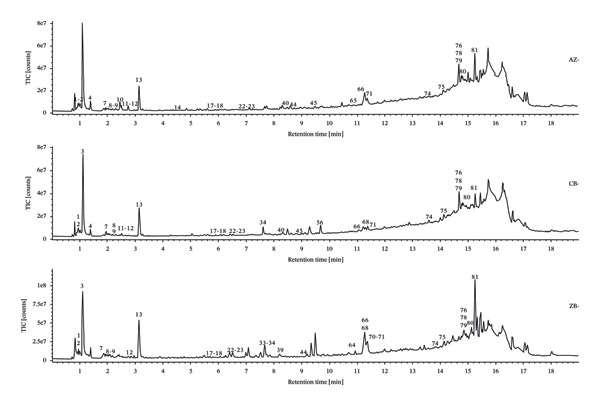
(b)

A total of 72 compounds were identified across all samples, including 44 alkaloids, 12 fatty acids, 4 nucleosides, 4 lignans, 3 sugars, 1 ketone, 1 fatty amide, 1 amide derivative, 1 amino acid, and 1 iridoid. Among these, 58 compounds were detected in *F. unibracteata*, 60 in *F. cirrhosa*, and 61 in *F. thunbergii*. In addition, 32 compounds were tentatively reported for the first time in the genus *Fritillaria* based on MS/MS fragmentation and database matching (Table 2).

**TABLE 2:** UPLC–Q‐TOF‐MS/MS analysis of positive‐ and negative‐ion modes of the three types of *Fritillaria*.

No.	t_Rpm_	Molecular formula	Component name	Compound type	AZ	CB	ZB
1	0.84	C_6_H_14_N_4_O_2_	Arginine^a^, ^c^	Amino acids	✓	✓	✓
2	1.00	C_12_H_22_O_11_	Sucrose^b^	Saccharides	✓	✓	✓
3	1.04	C_18_H_32_O_16_	Raffinose^a^, ^c^	Saccharides	✓	✓	✓
4	1.11	C_12_H_22_O_11_	β‐D‐Glu4‐1β‐D‐gal^b^	Saccharides	✓	✓	✓
5	1.31	C_4_H_6_O_4_	Succinic acid^b^, ^c^	Fatty acid	✓	✓	✓
6	1.95	C_4_H_4_N_2_O_2_	Uracil^a^	Nucleosides	✓	✓	✓
7	1.95	C_9_H_12_N_2_O_6_	Uridine^b^	Nucleosides	✓	✓	✓
8	2.02	C_10_H_13_N_5_O_4_	Adenosine^a^, ^b^	Nucleosides	✓	✓	✓
9	2.08	C_10_H_13_N_5_O_5_	Guanosine^a^, ^b^	Nucleosides	✓	✓	✓
10	2.46	C_6_H_9_NO_3_	Trimethadione^b^, ^c^, ^1^, ^2^	Ketone	✓	/	/
11	2.51	C_8_H_9_N	Indoline^a^, ^b^, ^c^	Heterocyclic derivatives of alkaloids	✓	✓	/
12	2.75	C_5_H_8_O_4_	Glutaric acid^b^, ^c^	fatty acid	✓	✓	✓
13	3.15	C_10_H_19_NO_5_	Hopantenic acid^a^, ^b^, ^c^	Acrylamide derivatives	✓	✓	✓
14	4.86	C_18_H_24_O_10_	Regaloside A^b^, ^c^	Iridoids	✓	/	/
15	5.48	C_33_H_55_NO_8_	Zhebeinine isomer^e^, ^a^	Isosteroidal alkaloids	✓	/	/
16	5.51	C_33_H_5_1NO_8_	Unidentified^e^, ^a^	Unidentified	✓	✓	✓
17	5.54 5.68	C_33_H_53_NO_8_	Edpetiline^a^, ^b^, ^d^	Isosteroidal alkaloids	✓	✓	✓
18	5.61	C_12_H_14_O_5_	(E)‐3,4,5‐Trimethoxycinnamic acid^b^, ^c^	Lignan	✓	✓	✓
19	5.66	C_27_H_41_NO_4_	3,11‐Dihydroxy‐5,6‐dihydro‐17,23‐epoxyveratraman‐6‐one^a^, ^c^, ^2^, ^3^	Isosteroidal alkaloids	✓	✓	✓
20	5.79	C_27_H_45_NO_4_	Verticine N‐oxide^a^	Isosteroidal alkaloid nitrogen oxides	/	/	✓
21	6.17	C_27_H_43_NO_4_	Unidentified^a^, ^e^, ^1^, ^3^	Unidentified	✓	✓	/
22	6.42 6.43	C_33_H_55_NO_8_	Zhebeininoside^a^, ^b^, ^1^, ^2^	Isosteroidal alkaloids	/	✓	✓
23	6.53 6.52	C_33_H_53_NO_8_	Hupehemonoside^a^, ^b^, ^1^, ^2^	Isosteroidal alkaloids	✓	✓	✓
24	6.65	C_27_H_45_NO_4_	CirrhosinineB^a^, ^1^	Steroid alkaloids	✓	✓	/
25	6.75	C_27_H_43_NO_3_	Sipeimine^a^, ^d^, ^1^, ^2^	Isosteroidal alkaloids	✓	✓	✓
26	6.92	C_27_H_43_NO_4_	Verticinone N‐oxide^a^	Isosteroidal alkaloid nitrogen oxides	/	/	✓
27	7.07	C_27_H_41_NO_3_	Peimisine^a^, ^d^, ^1^, ^2^	Isosteroidal alkaloids	✓	✓	✓
28	7.23	C_27_H_43_NO_4_	Unidentified^a^, ^e^	Unidentified	/	/	✓
29	7.24	C_33_H_51_NO_7_	Cycloposine^a^, ^c^, ^1^, ^3^	Isosteroidal alkaloids	/	✓	✓
30	7.34	C_27_H_41_NO_3_	(3beta,12alpha)‐3,12‐Dihydroxysolanid‐5‐en‐1‐one^a^, ^c^	Steroid alkaloids	✓	✓	✓
31	7.39	C_27_H_43_NO_2_	Delarinone^a^, ^2^	Isosteroidal alkaloids	/	✓	✓
32	7.40	C_27_H_45_NO_3_	Peimine^a^, ^d^, ^1^, ^2^, ^3^	Isosteroidal alkaloids	✓	✓	✓
33	7.53	C_33_H_40_O_18_	Unidentified^b^, ^e^, ^2^	Unidentified	/	/	✓
34	7.68	C_32_H_38_O_17_	6‐[(1 S,3aR,4 S,6aR)‐4‐(1,3‐Benzodioxol‐5‐yl)tetrahydro‐1H,3H‐furo[3,4‐c]furan‐1‐yl]‐1,3‐benzodioxol‐5‐yl 2‐O‐beta‐D‐glucopyranosyl‐beta‐D‐glucopyranoside^b^, ^c^, ^1^, ^2^, ^3^	Lignan	✓	/	✓
35	7.84	C_27_H_41_NO_2_	Cyclopamine isomer^b^, ^e^, ^1^, ^2^, ^3^	Isosteroidal alkaloids	/	✓	✓
36	7.85	C_27_H_43_NO_3_	Peiminine^a^, ^d^, ^1^, ^2^	Isosteroidal alkaloids	✓	✓	✓
37	8.06	C_27_H_39_NO_2_	Eratramine^a^, ^c^	Isosteroidal alkaloids	✓	✓	✓
38	8.16	C_27_H_37_NO_2_	N‐(9H‐Fluoren‐2‐yl)‐N[M‐H]‐hydroxytetradecanamide^a^, ^b^, ^c^, ^1^, ^3^	Fatty amides	/	✓	/
39	8.22	C_33_H_53_NO_8_	Hupehemonoside isomer^a^, ^b^, ^e^, ^2^, ^3^	Isosteroidal alkaloids	/	/	✓
40	8.34 8.33	C_39_H_63_NO_11_	Spirosol‐5‐en‐3‐ol‐4‐O‐α‐L‐rhamnopyranosyl‐(1–6) ‐β‐D‐glucopyranoside isomer^a^, ^b^, ^e^, ^2^, ^3^	Steroid alkaloids	✓	✓	/
41	8.43	C_33_H_55_NO_7_	Hupeheninoside^a^	Isosteroidal alkaloids	✓	✓	✓
42	8.46	C_27_H_45_NO_2_	Delarine^a^	Isosteroidal alkaloids	/	✓	/
43	8.50	C_27_H_41_NO_3_	Hupebenisine^a^	Isosteroidal alkaloids	✓	✓	✓
44	8.62 8.61	C_33_H_53_NO_7_	Solasodine 3‐β‐D‐glucopyranoside^a^, ^b^, ^c^, ^1^, ^2^	Steroid alkaloids	✓	/	✓
45	8.80	C_27_H_41_NO_2_	Cyclopamine^a^, ^c^, ^3^	Isosteroidal alkaloids	/	✓	✓
46	8.98	C_27_H_45_NO_2_	Petilidine^a^	Isosteroidal alkaloids	✓	✓	✓
47	9.00 9.01	C_36_H_65_N_7_O_10_	Unidentified^a^, ^b^, ^e^, ^1^, ^3^	Unidentified	/	✓	/
48	9.04	C_27_H_45_NO_3_	Zhebeinine^a^, ^2^, ^3^	Isosteroidal Alkaloids	/	/	✓
49	9.05	C_27_H_43_NO_2_	Zhebeirine^a^, ^b^, ^1^, ^3^	Isosteroidal alkaloids	✓	✓	✓
50	9.09 9.08	C_45_H_73_NO_15_	Solanidine 3‐O‐α‐L‐rhamnopyranosyl(1 ⟶ 2)‐[β‐D‐glucopyranosy‐(1 ⟶ 4)]‐β‐D‐glucopyranoside^a^, ^b^, ^c^	Steroid alkaloids	✓	✓	/
51	9.28	C_33_H_53_NO_6_	(3beta)‐Solanid‐5‐en‐3‐yl beta‐D‐glucopyranoside^a^, ^c^, ^2^	Steroid alkaloids	✓	✓	/
52	9.32	C_39_H_65_NO_11_	Demisidinoside^a^, ^1^, ^2^, ^3^	Steroid alkaloids	✓	/	/
53	9.35	C_33_H_55_NO_7_	Verticine 3‐glucoside^a^	Isosteroidal alkaloids	✓	✓	✓
54	9.52	C_28_H_45_NO_2_	Unidentified^a^, ^e^	Unidentified	/	/	✓
55	9.61	C_27_H_43_NO_2_	Ebeiedinone^a^	Isosteroidal alkaloids	✓	✓	✓
56	9.68 9.69	C_39_H_63_NO_11_	Spirosol‐5‐en‐3‐ol‐4‐O‐α‐L‐rhamnopyranosyl‐(1–6)‐β‐D‐glucopyranoside^a^, ^b^, ^c^, ^1^, ^3^	Steroid alkaloids	/	✓	/
57	9.90	C_27_H_43_NO_2_	Hupehenizine^a^, ^c^, ^1^, ^3^	Isosteroidal alkaloids	✓	✓	✓
58	9.91	C_28_H_45_NO_3_	Puqienine B^a^, ^1^, ^3^	Isosteroidal alkaloids	/	✓	/
59	10.00	C_27_H_43_NO_3_	Zhebeinone^a^, ^1^, ^2^, ^3^	Isosteroidal alkaloids	✓	/	✓
60	10.03	C_29_H_45_NO_3_	Spirosol‐5‐en‐3‐yl acetate^a^, ^c^	Steroid alkaloids	/	/	✓
61	10.43	C_28_H_47_NO_2_	Ningpeisine^a^	Isosteroidal alkaloids	✓	/	/
62	10.49	C_27_H_45_NO_2_	Ebedine^a^, ^3^	Isosteroidal alkaloids	✓	/	✓
63	10.83	C_27_H_43_NO_2_	Cordiline^a^, ^c^	Isosteroidal alkaloids	/	/	✓
64	10.94	C_33_H_52_O_10_	Unidentified^b^, ^e^, ^2^	Unidentified	/	/	✓
65	11.15	C_18_H_36_O_5_	9,10,18‐Trihydroxyoctadecanoic acid^b^, ^c^	Fatty acids	✓	/	/
66	11.23	C_22_H_22_O_8_	Picropodophyllin^b^	Lignan	✓	✓	✓
67	11.28	C_27_H_43_NO	17‐[1‐(5‐Methyl‐3,4,5,6‐tetrahydro‐2‐pyridinyl)ethyl]androst‐5‐en‐3‐ol^a^, ^c^	Steroid alkaloids	✓	✓	✓
68	11.29	C_18_H_34_O_5_	Pinellic acid^b^, ^1^, ^3^	Fatty acids	✓	✓	✓
69	11.36	C_27_H_45_NO	Demissidine^a^	Steroid alkaloids	✓	✓	/
70	11.36	C_35_H_52_O_11_	Unidentified^b^, ^e^	Unidentified	/	/	✓
71	11.39	C_23_H_24_O_9_	5‐Methoxypodophyllotoxin^b^	Lignan	✓	✓	✓
72	11.52	C_27_H_43_NO	Solanidine^a^, ^1^, ^3^	Steroid alkaloids	/	✓	/
73	11.53	C_28_H_49_NO_2_	Unidentified^a^, ^e^, ^1^	Unidentified	/	✓	/
74	13.60	C_18_H_34_O_4_	Dibutyl quinoate^b^, ^c^, ^1^	Fatty acids	✓	✓	✓
75	14.16	C_18_H_34_O_4_	Octadecanedioic acid^b^, ^c^	Fatty acids	✓	✓	✓
76	14.69	C_18_H_32_O_4_	(9E)‐Octadecylic acid^b^, ^c^, ^1^, ^2^, ^3^	Fatty acids	✓	✓	✓
77	14.75	C_19_H_38_O_4_	Monopalmitin^b^	Fatty acids	✓	✓	✓
78	14.79 14.83	C_18_H_30_O_3_	(9E,11E)‐13‐Oxooctadecadienoic acid^a^, ^b^, ^c^	Fatty acids	✓	✓	✓
79	14.79	C_18_H_32_O_4_	13‐Hydroperoxy‐9(E),11(E)‐octadecenoic acid^b^, ^c^	Fatty acids	✓	✓	✓
80	14.91 14.91	C_18_H_30_O_3_	(9Z,11 E)‐13‐Oxooctadecadienoic acid^a^, ^b^, ^c^, ^1^	Fatty acids	✓	✓	✓
81	15.27 15.47	C_18_H_32_O_3_	9‐Hydroxy‐10(E),12(Z)‐octadecadienoic acid^a^, ^b^, ^c^, ^1^, ^3^	Fatty acids	✓	✓	✓

*Note:* “✓” represents the presence of the compound; “/” indicates that the compound does not exist. Tentatively identified compounds were annotated based on MS/MS fragmentation patterns and database matching without reference standards.

^a^Represents detection in positive‐ion mode.

^b^Represents detection in negative‐ion mode.

^c^ The representative has been reported for the first time in this genus, tentatively identified by MS/MS and database matching.

^d^ is confirmed by comparison with the reference substance.

^e^ Representative tentatively identified.

^1^Represents the differential compounds between *F. unibracteata* and *F. cirrhosa*.

^2^Represents the differential compounds between *F. unibracteata* and *F. thunbergii*.

^3^Represents the differential compounds between *F. thunbergii* and *F. cirrhosa*; “‐” represents no such data.

Alkaloids, fatty acids, and nucleoside compounds constitute the major characteristic components of *Fritillaria* species [12]. As shown in Table 2 and Table S1, a total of 44 alkaloids, 12 fatty acids, and 4 nucleosides were identified in this study. The identified alkaloids included both heterosteroidal and steroidal alkaloids, whose structural verification was based on differences in carbon skeleton cleavage, side‐chain cleavage, and upper‐ring fragmentation patterns [28].

The characteristic fragment ions of heterosteroidal alkaloids (compounds 15, 17, 19, 20, 22, 23, 25, 27, 29, 31, 32, 35, 36, 37, 39, 41–43, 45, 46, 48, 49, 53, 55, 57–59, and 61–63) included m/z 147, 135, 121, and 107. The ion at m/z 147 is a cyclic ion generated by cleavage of the C–D ring with the loss of a vinyl group. The ion at m/z 135 is produced by cleavage between C4 and C5 of the C–D ring, while the m/z 121 ion results from breaking the C2–C3 bond. The fragment at m/z 107 is a stable ion formed by the cleavage of the butyl side chain outside the ring system [29].

The fragmentation of steroidal alkaloids (compounds 24, 26, 30, 40, 44, 50, 51, 52, 56, 60, 67, 69, and 72) typically involves the cleavage of C–C bonds both within and outside the ring system [30]. Their characteristic fragment ions include m/z 217, 189, 161, and 133. The ion at m/z 217 is formed by dehydration followed by the loss of a butyl side chain. The m/z 189 ion is a cyclic ion generated after removal of the cis‐propadiene acyl group from outside the ring. The ion at m/z 161 originates from cleavage of the C–C bond between rings A and *D*, whereas the ion at m/z 133 is a stable fragment formed after the removal of the ketone group from ring B [31].

A total of 20 fatty acid compounds (compounds 5, 12, 65, 68, and 74–81) were identified from the three *Fritillaria* species. Fatty acids often exist as positional isomers formed by variations in the locations of hydroxyl groups or double bonds. These compounds typically undergo cleavage at the carboxyl group, producing characteristic fragment ions. In addition, the presence of multiple unsaturated bonds makes fatty acids prone to McLafferty rearrangement [32].

For example, in compound 78, the loss of a water molecule (H_2_O) at the carboxyl group generates the fragment ion at m/z 275.3621 ([*M* + *H* − H_2_O]^+^). Subsequently, a McLafferty rearrangement leads to the formation of the fragment ion at m/z 219.0874 ([*M* + *H* − H_2_O − C_3_H_4_O]^+^). Based on comparison with previously reported data [33], compound 78 was identified as 13‐carbonyl‐9E,11E‐octadecadienoic acid.

A total of four nucleosides were identified from the *Fritillaria* samples (compounds 6–9). As nitrogen‐containing compounds, nucleosides readily produce [*M* + *H*]^+^ quasi‐molecular ions or [2M + *H*]^+^ adduct ions in positive‐ion mode during primary MS analysis. In MS^2^ spectra, nucleosides commonly undergo the loss of ribose (C_5_H_8_O_4_) or deoxyribose (C_5_H_8_O_3_), generating the characteristic base ion [*B* + *H*]^+^. Depending on the structure of the nucleobase, the [*B* + *H*]^+^ ion may subsequently lose neutral molecules such as NH_3_, H_2_O, HCN, CO, NH_2_CN, HNCO, or CNCHO, producing fragment ions including [*B* + *H* − 17]^+^, [*B* + *H* − 18]^+^, [*B* + *H* − 27]^+^, [*B* + *H* − 28]^+^, [*B* + *H* − 42]^+^, [*B* + *H* − 43]^+^, and [*B* + *H* − 55]^+^ [34].

For example, compound 9 exhibited a quasi‐molecular ion at m/z 284.0849 ([*M* + *H* − 132]^+^, C_5_H_6_N_5_O) in the MS^1^ spectrum, corresponding to the loss of a ribose unit. In the MS^2^ spectrum, the resulting fragment at m/z 152.0571 ([*M* + *H* − 132]^+^, C_5_H_3_N_4_O) was formed by ribose loss, followed by further neutral losses such as NH_3_ and NH_2_CN, yielding fragment ions at m/z 135.0921 ([*M* + *H* − 147]^+^, C_5_H_3_N_4_O) and m/z 110.0346 ([*M* + *H* − 172]^+^, C_4_H_4_N_3_O), respectively. Based on comparison with previously reported data [35], compound 9 was identified as guanosine.

A total of four lignans (compounds 18, 34, 66, and 71) were identified from the *Fritillaria* samples. Their main fragmentation pathways include *β*‐allyl cleavage, biphenyl cleavage, ester bond cleavage, and alkyl side‐chain cleavage [36]. Lignans typically exhibit high sensitivity and strong signal responses in negative‐ion mode. As a result, they readily undergo neutral losses such as H_2_O, CH_3_, and CH_2_O, producing characteristic fragment ions including [M − H_2_O]^-^, [M − CH_2_O]^-^, and [M − CH_3_ − H]^-^ [37].

A total of three sugar components (compounds 2–4) were identified from the *Fritillaria* samples. In most sugars, ketoses and aldoses readily undergo ring‐opening reactions under alkaline conditions, generating acidic ions and glycosyl fragments. These cleavages generally occur through keto–enol isomerization and hydroxyl dehydration. In MS^2^ spectra, sugar molecules typically undergo cleavage at aldehyde or ketone groups, whereas aldehyde groups may further experience oxidation and dehydration. As a result, characteristic fragment ions such as m/z 29.0453 ([M − CHO]^-^) and m/z 43.0428 ([M − CO − CH_3_]^-^) are produced [38].

One ketone compound (compound 10) was also identified. In this compound, a methyl group is removed to form a radical species, which subsequently undergoes further cleavage, including C–O bond dissociation, producing CO and generating new radical intermediates. Characteristic fragments such as m/z 116.0638, 87.0253, and 71.0294 were observed. Based on comparison with published reports [39], compound 10 was tentatively identified as trimethadione.

In addition, one aliphatic amide (compound 38), one amide derivative (compound 13), one amino acid (compound 1), and one iridoid compound (compound 14) were identified. Amides and their derivatives generally undergo amide‐bond cleavage during fragmentation, resulting in dissociation of the amide moiety. Their acyl or imine groups may also undergo fragmentation, producing characteristic ions such as m/z 104.0713 and 86.0608 [40].

The fragmentation behavior of amino acids commonly involves loss of imine groups, side‐chain cleavage, and carbon skeleton dissociation [41]. Compound 1 first undergoes loss of an amino group, followed by the loss of a guanidino group from the side chain, consistent with a fragmentation pathway of [M − NH_2_ − 3NH_3_]^+^.

Iridoids possess a characteristic C_10_ skeleton, in which the C4 position is typically substituted with methyl, carboxyl, methyl ester, or hydroxymethyl groups. However, iridoid structures are relatively unstable. Under low‐energy conditions, initial dehydrogenation produces the [M−H]^-^ quasi‐molecular ion. Under higher‐energy conditions, functional groups on the parent ring are readily lost, including neutral species such as H_2_O, CO_2_, CH_3_OH, CH_3_COOH, and sugar moieties. Based on comparison with previously reported data [42], compound 14 was tentatively identified as regaloside A.

### 3.3. PCA Analysis

The TIC profiles of the three *Fritillaria* species showed clear differences in their chemical compositions. Therefore, PCA was performed separately for the positive‐ and negative‐ion datasets to evaluate overall metabolic trends (Figure 2). In both ionization modes, distinct clustering patterns were observed among the three *Fritillaria* species, indicating significant differences in their metabolite profiles.

FIGURE 2PCA diagram of positive‐ion (a) and negative‐ion (b) modes in three *Fritillaria* spp.

**Figure:**
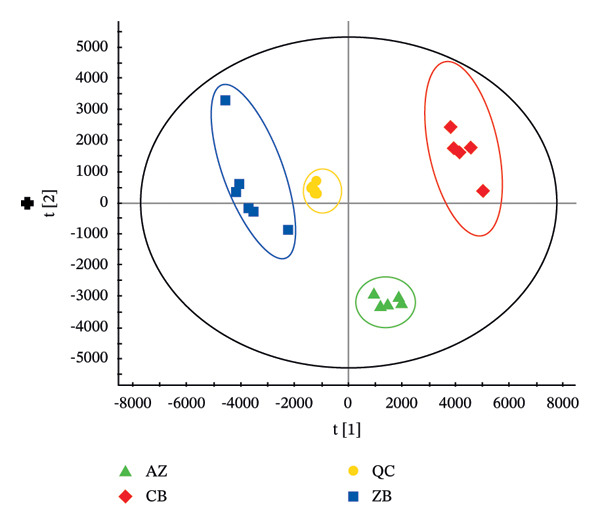
(a)

**Figure:**
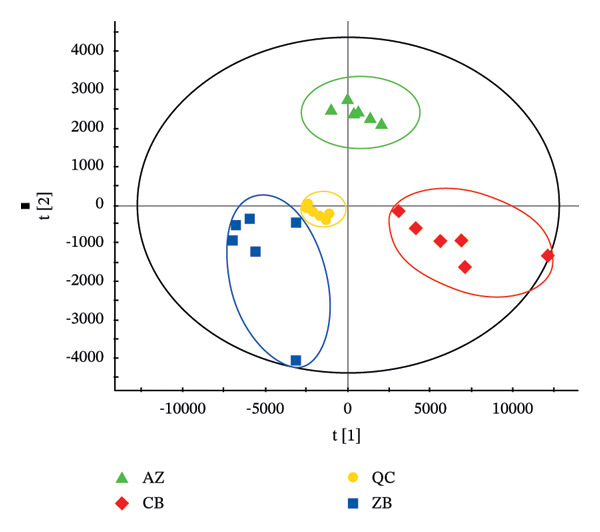
(b)

### 3.4. Differential Chemical Composition Analysis

Based on the TIC and PCA results, OPLS‐DA was performed to further characterize differences among the three *Fritillaria* species. Clear separation among *F. unibracteata*, *F. cirrhosa*, and *F. thunbergii* was observed in both positive‐ and negative‐ion modes (Figures 3(a), 3(c), and 3(e)). Differential ions were screened by integrating the S‐plot derived from the OPLS‐DA model with relatively stringent VIP thresholds of > 5 in positive‐ion mode and > 4 in negative‐ion mode to highlight metabolites with stronger discriminatory power (Figures 3(b), 3(d), and 3(f)).

FIGURE 3S‐plots in OPLS‐DA model (a, c, e) and PCA score plots (b, d, f) of *F. unibracteata*, *F. cirrhosa,* and *F. thunbergii*.

**Figure:**
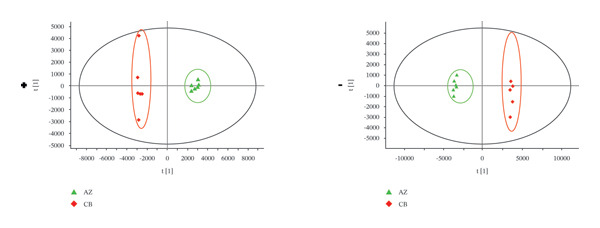
(a)

**Figure:**
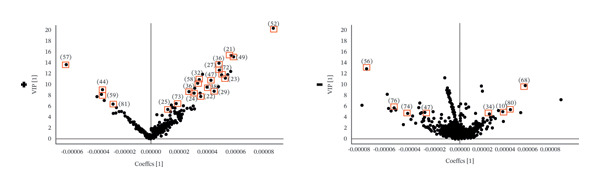
(b)

**Figure:**
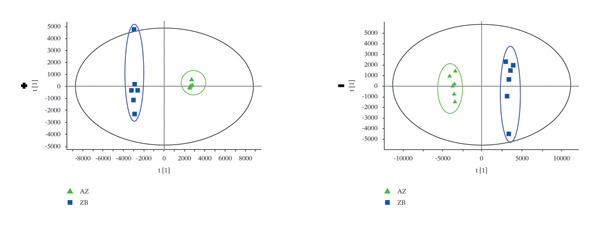
(c)

**Figure:**
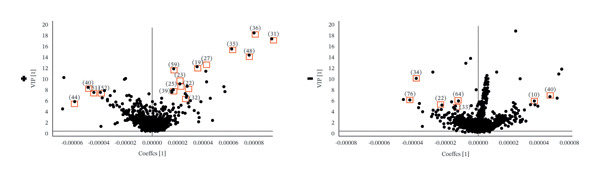
(d)

**Figure:**
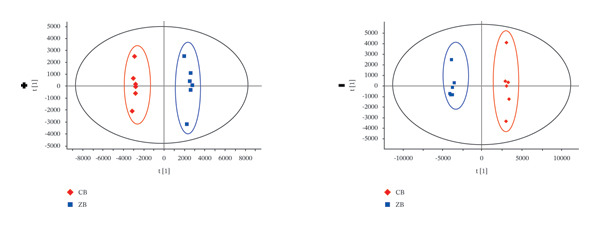
(e)

**Figure:**
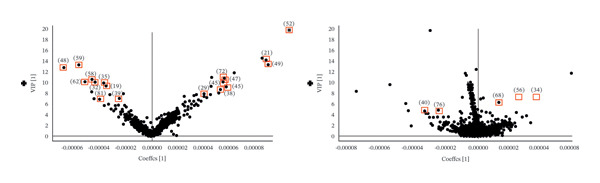
(f)

A total of 28 differential metabolites were identified between *F. unibracteata* and *F. cirrhosa*, including zhebeininoside, hupehemonoside, fritimine B, sipeimine, peimisine, cycloposine, peimine, and peiminine. Between *F. unibracteata* and *F. thunbergii*, 22 differential compounds were detected, such as hupehemonoside, sipeimine, peimisine, delarinone, peimine, and peiminine. Additionally, 23 differential metabolites were identified between *F. thunbergii* and *F. cirrhosa*, including peimine, zhebeirine, demissidinoside, hupehenizine, puqienine B, zhebeinone, ebedine, and solanidine. The chemical structures of all identified differential compounds are shown in Figure S1.

In addition, based on the mean relative abundances of the differential metabolites (Figure 4), we found that only 12 compounds—such as solasodine 3‐β‐D‐glucopyranoside, hupehenizine, zhebeinone, and trimethadione—exhibited higher levels in *F. unibracteata* compared with *F. cirrhosa* and *F. thunbergii*.

FIGURE 4The differential compounds between *F. unibracteata* and *F. cirrhosa* (a); (b) the differential compounds between *F. unibracteata* and *F. thunbergii*; (c) the differential compounds between *F. cirrhosa* and *F. thunbergii*. The *x*‐axis represents species, while the *y*‐axis represents the relative abundance values of differential compounds.

**Figure:**
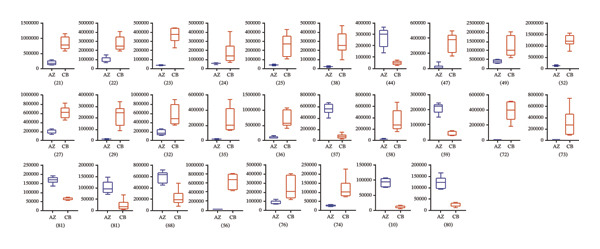
(a)

**Figure:**
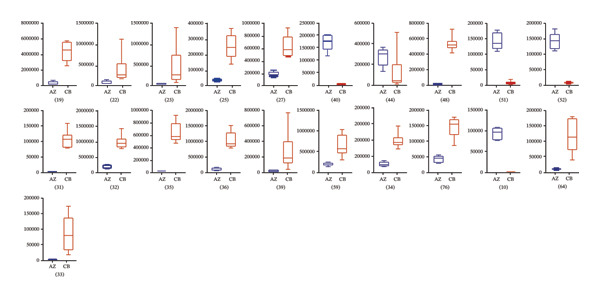
(b)

**Figure:**
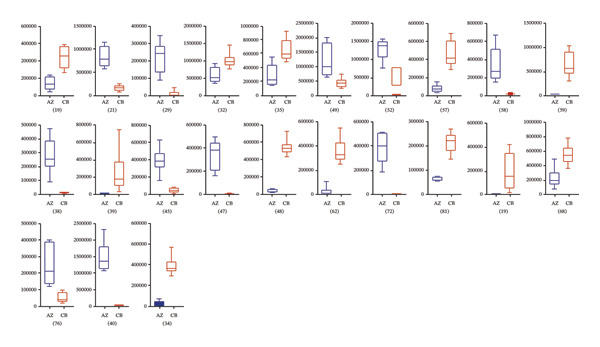
(c)

When comparing *F. cirrhosa* and *F. thunbergii*, the distribution patterns of differential compound abundances were relatively balanced. Eleven metabolites, including cycloposine and cyclopamine, were more abundant in *F. cirrhosa*, whereas twelve metabolites—such as zhebeinine, hupehenizine, zhebeinone, ebedine, and pinellic acid—were present at higher levels in *F. thunbergii*.

## 4. Discussion

In this study, a total of 72 chemical constituents were characterized across three *Fritillaria* species, including alkaloids, fatty acids, nucleosides, and other minor compound classes. Among these, steroidal alkaloids such as peimine, peiminine, and sipeimine are widely regarded as major bioactive components of *Fritillaria* and have been reported to exert antitussive and antiasthmatic effects through suppression of the cough center and modulation of oxidative stress and inflammatory mediators, including malondialdehyde (MDA), interleukins (IL‐1, IL‐6, and IL‐8), and hypoxia‐inducible factor‐1*α* (HIF‐1*α*) [43–46]. The detection of these representative alkaloids in the investigated species is consistent with previous pharmacological findings and supports their relevance for respiratory‐related therapeutic applications [43].

Beyond alkaloids, several fatty acid derivatives, including succinic acid, glutaric acid, and pinellic acid, were detected. These compounds have been associated with sedative, hypotensive, and spasmolytic effects and may contribute to protective roles under hypoxic stress and in neurological disorders [45–47]. In addition, nucleosides such as adenosine, guanosine, and inosine are known to participate in cardiovascular regulation and neuromodulation via platelet aggregation inhibition, coronary vasodilation, smooth muscle relaxation, and central nervous system sedation [47, 48]. Collectively, the co‐occurrence of these compound classes indicates a multicomponent chemical basis that may underlie the diverse pharmacological properties of *Fritillaria* medicinal materials.

Notably, succinic acid was tentatively annotated in *Fritillaria* in this work and, to our knowledge, has not been previously reported in the genus based on available literature. Previous studies have described its sedative, anticonvulsant, and analgesic activities, potentially through modulation of neuronal excitability and inflammatory responses [49–51]. This observation expands the currently reported chemical diversity of the genus and may provide a supplementary phytochemical clue for differentiation; however, its diagnostic relevance requires further validation through structural confirmation and expanded sampling.

The metabolomic profiles revealed clear interspecific differences in both qualitative and quantitative composition. The differential compounds between *F. unibracteata* and the other two species were mainly heterosteroidal alkaloids, including sipeimine, peimisine, and peiminine. Heterosteroidal alkaloid abundance is commonly used as an important criterion for quality evaluation of *Fritillaria* medicinal materials [52–54], which aligns with the reported pharmacological potency of *F. unibracteata* [55, 56]. In addition, trimethadione was uniquely detected in *F. unibracteata*, suggesting its potential utility as a species‐associated chemical feature for discrimination [57].

Compared with *F. unibracteata*, *F. cirrhosa* exhibited a more diverse differential profile, including steroidal alkaloids and fatty acid‐related constituents such as solanidine and demissidine, whereas *F. thunbergii* showed characteristic components including ribinin and ribinone. These compositional distinctions provide a chemical basis that may partially explain differences in potency among species and support the feasibility of species discrimination based on metabolite patterns.

From a chemotaxonomic perspective, it is informative to interpret LC–MS‐derived metabolite signatures alongside prior multievidence discrimination strategies. A recent study achieved robust differentiation of *Fritillaria* species by integrating morphometric traits with GC–MS‐based chemical profiling, highlighting the value of combining morphological and chemical markers for species authentication and interpretability [58]. In this context, the species‐characteristic metabolite patterns identified by UPLC–Q‐TOF‐MS/MS in the present work can be regarded as complementary chemotaxonomic evidence; future integration of targeted metabolite markers with morphology‐based identification may further strengthen authentication and quality control.

It should also be noted that *F. unibracteata* and *F. cirrhosa* samples were collected from natural habitats, whereas *F. thunbergii* was obtained from a commercial source. Differences in origin, cultivation conditions, environmental factors, and post‐harvest processing may influence metabolite accumulation. Therefore, part of the observed variability could reflect ecological or cultivation‐related effects rather than purely species‐driven differences, and expanded sampling across multiple origins would help disentangle these effects in future studies.

From the resource utilization perspective, the limited distribution of *F. unibracteata* and *F. cirrhosa*, together with increasing demand for high‐quality medicinal materials, has intensified harvesting pressure on wild populations in the Qinghai–Tibetan Plateau. Their slow reproduction and regeneration further constrain population recovery. In contrast, *F. thunbergii* is widely used and commercially important, with a substantially larger market share [12, 59]. The identification of shared bioactive constituents and species‐characteristic metabolites suggests that cultivated resources (e.g., *F. thunbergii*) may partially meet medicinal demand through rational utilization of overlapping active components, while marker‐oriented authentication may support graded quality evaluation and reduce pressure on wild resources.

Overall, despite compositional differences, all three species contained pharmacologically relevant constituents such as peimine and demissidinoside. Further work combining deeper constituent isolation, structural confirmation, and mechanism‐oriented pharmacological validation will clarify which metabolite classes drive efficacy differences, facilitate the establishment of robust quality evaluation systems, and support sustainable utilization while contributing to the conservation of wild and endangered *Fritillaria* resources.

## 5. Conclusions

This study employed UPLC–Q‐TOF‐MS/MS to comprehensively characterize and compare the chemical profiles of three *Fritillaria* species. In total, 44 alkaloids, 12 fatty acids, 4 nucleosides, 4 lignans, 3 sugars, and several minor compound classes were annotated, and 32 constituents were newly reported within the *Fritillaria* genus. Multivariate analysis further demonstrated clear qualitative and quantitative differences among species. Using OPLS‐DA‐based screening, 28 differential metabolites were identified between *F*. *unibracteata* and *F. cirrhosa*, 22 between *F. unibracteata* and *F. thunbergii*, and 23 between *F. thunbergii* and *F. cirrhosa*. These newly reported constituents expand the chemical space of the genus and provide additional candidate features for marker‐oriented authentication and quality evaluation.

These differential metabolites provide candidate chemical markers for species discrimination and offer supporting evidence for improving authentication and quality evaluation of *Fritillaria* medicinal materials. Overall, the findings expand current knowledge of chemical diversity in the genus and provide a phytochemical basis for developing more comprehensive quality assessment strategies and for promoting sustainable utilization and conservation of valuable *Fritillaria* resources.

## Funding

This work was supported by the Key Laboratory Project of Medicinal Animal and Plant Resources in Qinghai–Tibet Plateau (Grant No. 2020‐YJ‐Y40), the National Positioning Research Station Project for Forest Ecosystem on the South Slope of Qilian Mountains (Grant Nos. 212, 712, and 127), the Kunlun Talent?High‐End Innovation and Entrepreneurship Talent Program Featured Talent Foundation of China (Grant No. 1003‐005024011), and the Formation Mechanism and Utilization Team of Characteristic Germplasm Resources in the Qinghai–Tibet Plateau (Grant No. QHKLYC‐GDCXCY‐2024‐597).

## Conflicts of Interest

The authors declare no conflicts of interest.

## Supporting Information

*Supporting 1.* Table S1: detailed information on the identified compounds in the three types of *Fritillaria* based on UPLC–Q‐TOF‐MS/MS analysis.

*Supporting 2.* Figure S1: structural formulas of compounds identified from *F. unibracteata, F. cirrhosa*, and *F. thunbergii.*

## Supporting information


**Supporting Information** Additional supporting information can be found online in the Supporting Information section.

## Data Availability

The data used to support the findings of this study are included within the article.
