# Supplementary material for: Analysis of Chemical Composition in Three Types of Fritillaria Using UPLC–Q‐TOF‐MS/MS Technology
Source: Int J Anal Chem. 2026 Apr 29;2026:9972411. doi: 10.1155/ianc/9972411 (PMC13126092; doi:10.1155/ianc/9972411)
Supplement: Supplementary file 1 — Supporting Information Additional supporting information can be found online in the Supporting Information section. [file IANC-2026-9972411-s001.docx]

# International Journal of Analytical Chemistry

# Analysis of Chemical Composition in Three Types of

# Fritillaria Using UPLC-Q-TOF-MS/MS Technology

Shuang Ji¹˒³˒⁴, Xufeng Mao¹˒³, Desheng Qi²˒³, Shaoxiong Zhang²˒³, Zhi Chen²˒³, Feng Qiao²˒³, Zhiqiang Dong¹, Shaobo Du¹, and Huichun Xie²˒³˒⁴*

¹ School of Geographic Sciences, Qinghai Normal University, Xining 810008, China
² School of Life Sciences, Qinghai Normal University, Xining 810008, China
³ Key Laboratory of Medicinal Animal and Plant Resources on the Qinghai–Tibet Plateau, Xining 810008, China
⁴ National Positioning Observation and Research Station for Forest Ecosystem on the South Slope of the Qilian Mountains in Qinghai, Huzhu 810500, China

*Corresponding author: Huichun Xie
Email: xiehuichun@qhnu.edu.cn

|  |
| --- |

**Table S1** Detail information of compounds in three types Fritillaria

| NO | *t*_Rpm_ | Molecular formula | Quasi molecular ion peak  m/z | Adduct ion | Error  ×10^-6^ | Fragmentation ion  m/z | Relative molecular mass (m/z)  measured value | Relative molecular mass (m/z)  theoretical value |
| --- | --- | --- | --- | --- | --- | --- | --- | --- |
| 1 | 0.84 | C_6_H_14_N_4_O_2_ | 175.119 7 | [M+H]^+^ | 4.5 | 70.065 8 | 174.112 5 | 174.111 7 |
| 2 | 1.00 | C_12_H_22_O_11_ | 387.114 7 | [M+HCOO]^-^ | 0.7 | 161.045 7 | 342.116 5 | 342.116 2 |
| 3 | 1.04 | C_18_H_32_O_16_ | 549.167 3 | [M+HCOO]^-^ | 0.2 | 267.073 0 | 504.169 1 | 504.169 0 |
| 4 | 1.11 | C_12_H_22_O_11_ | 387.114 9 | [M+HCOO]^-^ | 1.2 | 179.016 9  161.045 4 | 342.116 7 | 342.116 2 |
| 5 | 1.31 | C_4_H_6_O_4_ | 163.025 3 | [M+HCOO]^-^ | 3.2 | 103.003 6 | 118.027 1 | 118.026 6 |
| 6 | 1.95 | C_4_H_4_N_2_O_2_ | 113.035 1 | [M+H]^+^ | 5.1 | 119.035 5  107.050 2 | 112.027 9 | 112.027 3 |
| 7 | 1.95 | C_9_H_12_N_2_O_6_ | 243.062 8 | [M-H]^-^ | 2.2 | 111.020 8 | 244.070 1 | 244.069 5 |
| 8 | 2.02 | C_10_H_13_N_5_O_4_ | 268.105 2 | [M+H]^+^ | 4.2 | 137.064 0  136.062 5  119.035 5  107.050 2 | 267.097 9 | 267.096 8 |
|  |  |  | 312.095 5 | [M+HCOO]^-^ | 1.8 | 134.047 6  117.019 7 | 267.097 3 | 267.096 8 |
| 9 | 2.08 | C_10_H_13_N_5_O_5_ | 284.084 9 | [M+H]^+^ | 2.9 | 152.057 1  135.029 1 | 283.092 5 | 283.091 7 |
|  |  |  | 282.221 3 | [M-H]^-^ | 1.7 | 150.042 2  133.015 5 | 283.092 1 | 283.091 7 |
| 10 | 2.46 | C_6_H_9_NO_3_ | 142.051 4 | [M-H]^-^ | 3.4 | 104.071 3 | 143.058 7 | 143.058 2 |
| 11 | 2.51 | C_8_H_9_N | 120.081 5 | [M+H]^+^ | 6.3 | 103.054 7  91.054 2  77.038 9 | 119.074 3 | 119.074 3 |
|  |  |  | 164.072 1 | [M+HCOO]^-^ | 2.4 | 103.055 4 | 119.073 9 | 119.073 5 |
| 12 | 2.75 | C_5_H_8_O_4_ | 131.035 4 | [M-H]^-^ | 3.6 | 87.045 2 | 132.042 7 | 132.042 3 |
| 13 | 3.15 | C_10_H_19_NO_5_ | 234.134 6 | [M+H]^+^ | 4.5 | 104.071 3  86.060 8 | 233.127 4 | 233.126 3 |
|  |  |  | 232.119 8 | [M-H]^-^ | 3.4 | 102.066 5 | 233.127 1 | 233.126 3 |
| 14 | 4.86 | C_18_H_24_O_10_ | 399.129 6 | [M-H]^-^ | -0.2 | 163.040 2  145.029 8  119.050 2 | 400.136 9 | 400.136 9 |
| 15 | 5.48 | C_33_H_55_NO_8_ | 594.399 1 | [M+H]^+^ | 0.6 | 103.055 4 | 593.391 8 | 593.391 4 |
| 16 | 5.51 | C_33_H_5_1NO_8_ | 590.367 9 | [M+H]^+^ | -1.4 | 145.029 8  119.050 2 | 589.360 6 | 589.361 5 |
| 17 | 5.54 | C_33_H_53_NO_8_ | 592.382 8 | [M+H]^+^ | -2.8 | 279.094 6 | 591.375 5 | 591.377 1 |
|  | 5.68 |  | 636.374 2 | [M+HCOO]^-^ | -1.8 | 149.045 6  89.023 8 | 591.376 0 | 591.377 1 |
| 18 | 5.61 | C_12_H_14_O_5_ | 237.077 5 | [M-H]^-^ | 2.6 | 145.029 9  117.035 1 | 238.084 7 | 238.084 1 |
| 19 | 5.66 | C_27_H_41_NO_4_ | 444.311 0 | [M+H]^+^ | 0.3 | 426.091 3  118.086 6  114.091 5 | 443.303 7 | 443.303 6 |
| 20 | 5.79 | C_27_H_45_NO_4_ | 448.342 0 | [M+H]^+^ | -0.4 | 126.128 2  118.086 8 | 447.334 7 | 447.334 9 |
| 21 | 6.17 | C_27_H_43_NO_4_ | 446.327 0 | [M+H]^+^ | 1.1 | 118.086 8  114.091 7 | 445.319 7 | 445.319 2 |
| 22 | 6.42 | C_33_H_55_NO_8_ | 594.399 4 | [M+H]^+^ | -1.1 | 177.055 4  145.029 1  139.133 4 | 593.392 1 | 593.392 8 |
|  | 6.43 |  | 638.391 0 | [M+HCOO]^-^ | 0.0 | 193.050 8  175.040 1  160.106 1 | 593.392 8 | 593.392 8 |
| 23 | 6.53 | C_33_H_53_NO_8_ | 592.383 9 | [M+H]^+^ | -0.9 | 414.336 8  118.086 4 | 591.376 6 | 591.377 1 |
|  | 6.52 |  | 636.373 5 | [M+HCOO]^-^ | -2.9 | 367.083 6  289.082 2  245.093 6  203.082 1 | 591.375 3 | 591.377 1 |
| 24 | 6.65 | C_27_H_45_NO_4_ | 448.342 6 | [M+H]^+^ | 1.0 | 118.086 8  114.091 7 | 447.335 3 | 447.334 9 |
| 25 | 6.75 | C_27_H_43_NO_3_ | 430.331 8 | [M+H]^+^ | 0.5 | 126.128 2  118.086 8 | 429.324 5 | 429.324 3 |
| 26 | 6.92 | C_27_H_43_NO_4_ | 448.342 3 | [M+H]^+^ | 0.3 | 112.112 8  98.096 9 | 447.335 0 | 447.334 9 |
| 27 | 7.07 | C_27_H_41_NO_3_ | 428.316 5 | [M+H]^+^ | 1.3 | 126.128 3  118.086 8  114.091 3  109.101 2  98.096 6 | 427.309 2 | 427.308 6 |
| 28 | 7.23 | C_27_H_43_NO_4_ | 446.327 4 | [M+H]^+^ | 2.0 | 112.112 8  98.096 9 | 445.319 2 | 445.319 2 |
| 29 | 7.24 | C_33_H_51_NO_7_ | 574.374 0 | [M+H]^+^ | 0.2 | 413.324 0  118.086 7 | 573.366 7 | 573.366 6 |
| 30 | 7.34 | C_27_H_41_NO_3_ | 428.316 2 | [M+H]^+^ | 0.6 | 118.087 1  98.097 0 | 427.308 9 | 427.308 6 |
| 31 | 7.39 | C_27_H_43_NO_2_ | 414.337 3 | [M+H]^+^ | 1.6 | 145.100 1  119.088 8  118.087 1  98.097 0  91.054 9 | 413.330 1 | 413.329 4 |
| 32 | 7.40 | C_27_H_45_NO_3_ | 432.347 5 | [M+H]^+^ | 0.6 | 308.223 1  118.086 8 | 431.340 2 | 431.339 9 |
| 33 | 7.53 | C_33_H_40_O_18_ | 723.213 8 | [M-H]^-^ | -0.5 | 124.112 3  118.086 9 | 724.221 1 | 724.221 5 |
| 34 | 7.68 | C_32_H_38_O_17_ | 693.202 8 | [M-H]^-^ | -1.2 | 177.055 4  145.029 1 | 694.210 1 | 694.210 9 |
| 35 | 7.84 | C_27_H_41_NO_2_ | 412.321 8 | [M+H]^+^ | 1.9 | 396.290 0  118.086 6  98.096 6 | 411.314 5 | 411.313 7 |
| 36 | 7.85 | C_27_H_43_NO_3_ | 430.332 1 | [M+H]^+^ | 1.2 | 118.086 9 | 429.324 8 | 429.324 3 |
| 37 | 8.06 | C_27_H_39_NO_2_ | 410.305 8 | [M+H]^+^ | 1.0 | 118.086 7 | 409.298 5 | 409.298 1 |
| 38 | 8.16 | C_27_H_37_NO_2_ | 408.290 8 | [M+H]^+^ | 2.7 | 139.113 2  127.113 0 | 407.283 5 | 407.282 4 |
| 39 | 8.22 | C_33_H_53_NO_8_ | 592.384 2 | [M+H]^+^ | -0.3 | 410.306 5  251.178 9  177.054 8  124.112 3  118.086 9 | 591.376 9 | 591.377 1 |
|  |  |  | 636.375 0 | [M+HCOO]^-^ | -0.5 | 175.039 7 | 591.376 8 | 591.377 1 |
| 40 | 8.34 | C_39_H_63_NO_11_ | 722.446 2 | [M+H]^+^ | -1.6 | 139.113 2  127.113 0 | 721.439 0 | 721.440 1 |
|  | 8.33 |  | 766.437 0 | [M+HCOO]^-^ | -1.7 | 119.088 8  118.087 1  98.097 0 | 721.438 8 | 721.440 1 |
| 41 | 8.43 | C_33_H_55_NO_7_ | 578.404 9 | [M+H]^+^ | -0.3 | 383.147 2  118.086 8 | 577.397 7 | 577.397 9 |
| 42 | 8.46 | C_27_H_45_NO_2_ | 416.353 0 | [M+H]^+^ | 1.7 | 177.055 4  145.029 1 | 415.345 7 | 415.345 0 |
| 43 | 8.50 | C_27_H_41_NO_3_ | 428.316 4 | [M+H]^+^ | 1.1 | 114.090 9 | 427.309 1 | 427.308 6 |
| 44 | 8.62 | C_33_H_53_NO_7_ | 576.389 7 | [M+H]^+^ | 0.4 | 414.337 9  398.341 5 | 575.382 4 | 575.382 2 |
|  | 8.61 |  | 620.380 2 | [M+HCOO]^-^ | -0.4 | 415.145 7 | 575.382 0 | 575.382 2 |
| 45 | 8.80 | C_27_H_41_NO_2_ | 412.321 9 | [M+H]^+^ | 2.2 | 396.290 0  118.087 0  98.096 6 | 411.314 6 | 411.313 7 |
| 46 | 8.98 | C_27_H_45_NO_2_ | 416.352 7 | [M+H]^+^ | 0.9 | 253.196 2  118.086 9  98.097 2 | 415.345 4 | 415.345 0 |
| 47 | 9.00 | C_36_H_65_N_7_O_10_ | 756.487 7 | [M+H]^+^ | 1.5 | 118.086 6  112.112 6 | 755.480 4 | 755.479 3 |
|  | 9.01 |  | 800.479 0 | [M+HCOO]^-^ | 1.9 | 124.112 3  118.086 9 | 755.480 8 | 755.479 3 |
| 48 | 9.04 | C_27_H_45_NO_3_ | 432.347 7 | [M+H]^+^ | 1.1 | 118.086 9  98.096 2 | 431.340 4 | 431.339 9 |
| 49 | 9.05 | C_27_H_43_NO_2_ | 414.337 2 | [M+H]^+^ | 1.3 | 118.086 9  98.096 2 | 413.329 9 | 413.329 4 |
| 50 | 9.09 | C_45_H_73_NO_15_ | 868.502 4 | [M+H]^+^ | -3.4 | 430.368 4  118.086 9 | 867.495 1 | 867.498 0 |
|  | 9.08 |  | 912.494 0 | [M+HCOO]^-^ | -2.4 | 429.119 7 | 867.495 8 | 867.498 0 |
| 51 | 9.28 | C_33_H_53_NO_6_ | 560.394 7 | [M+H]^+^ | 0.2 | 398.342 4 | 559.387 4 | 559.387 3 |
| 52 | 9.32 | C_39_H_65_NO_11_ | 724.461 2 | [M+H]^+^ | -2.5 | 508.319 0 | 723.453 9 | 723.455 8 |
| 53 | 9.35 | C_33_H_55_NO_7_ | 578.404 6 | [M+H]^+^ | -1.0 | 177.055 5  119.050 4 | 577.397 3 | 577.397 9 |
| 54 | 9.52 | C_28_H_45_NO_2_ | 428.352 3 | [M+H]^+^ | -0.1 | 119.088 8  118.087 1  98.097 0 | 427.345 0 | 427.345 0 |
| 55 | 9.61 | C_27_H_43_NO_2_ | 414.337 5 | [M+H]^+^ | 2.1 | 118.086 8 | 413.330 3 | 413.329 4 |
| 56 | 9.68 | C_39_H_63_NO_11_ | 722.446 4 | [M+H]^+^ | -1.4 | 560.394 6  398.342 9 | 721.439 1 | 721.440 1 |
|  | 9.69 |  | 766.437 3 | [M+HCOO]^-^ | -1.3 | 558.379 4 | 721.439 1 | 721.440 1 |
| 57 | 9.90 | C_27_H_43_NO_2_ | 414.337 5 | [M+H]^+^ | 2.1 | 118.087 0 | 413.330 2 | 413.329 4 |
| 58 | 9.91 | C_28_H_45_NO_3_ | 444.347 8 | [M+H]^+^ | 1.3 | 118.086 6  112.112 6  81.069 9 | 443.340 5 | 443.339 9 |
| 59 | 10.00 | C_27_H_43_NO_3_ | 430.332 1 | [M+H]^+^ | 2.1 | 111.104 5 | 429.325 2 | 429.324 3 |
| 60 | 10.03 | C_29_H_45_NO_3_ | 456.347 6 | [M+H]^+^ | 0.9 | 126.128 1  112.112 5 | 455.340 4 | 455.339 9 |
| 61 | 10.43 | C_28_H_47_NO_2_ | 430.368 3 | [M+H]^+^ | 0.8 | 118.087 0  95.085 7 | 429.361 0 | 429.360 7 |
| 62 | 10.49 | C_27_H_45_NO_2_ | 416.352 9 | [M+H]^+^ | 1.4 | 177.055 4 | 415.345 6 | 415.345 0 |
| 63 | 10.83 | C_27_H_43_NO_2_ | 414.336 9 | [M+H]^+^ | 0.6 | 177.055 3  118.086 8  98.097 2 | 413.329 6 | 413.329 4 |
| 64 | 10.94 | C_33_H_52_O_10_ | 607.348 8 | [M-H]^-^ | 0.1 | 119.088 8  118.087 1 | 608.356 1 | 608.356 0 |
| 65 | 11.15 | C_18_H_36_O_5_ | 331.249 4 | [M-H]^-^ | 1.3 | 177.054 8  124.112 3  118.086 9 | 332.256 7 | 332.256 3 |
| 66 | 11.23 | C_22_H_22_O_8_ | 413.124 8 | [M-H]^-^ | 1.4 | 193.051 1  177.055 5  119.050 4 | 414.132 1 | 414.131 5 |
| 67 | 11.28 | C_27_H_43_NO | 398.342 4 | [M+H]^+^ | 1.7 | 126.127 7  98.096 4 | 397.335 2 | 397.334 5 |
| 68 | 11.29 | C_18_H_34_O_5_ | 329.234 2 | [M-H]^-^ | 2.6 | 171.103 1 | 330.241 5 | 330.240 6 |
| 69 | 11.36 | C_27_H_45_NO | 400.357 3 | [M+H]^+^ | -0.3 | 139.113 2  127.113 0 | 399.350 0 | 399.350 1 |
| 70 | 11.36 | C_35_H_52_O_11_ | 693.348 6 | [M+HCOO]^-^ | -0.9 | 139.113 2  127.113 0 | 648.350 4 | 648.351 0 |
| 71 | 11.39 | C_23_H_24_O_9_ | 443.134 9 | [M-H]^-^ | 0.3 | 171.102 9  139.113 2  127.113 0 | 444.142 2 | 444.142 0 |
| 72 | 11.52 | C_27_H_43_NO | 398.342 5 | [M+H]^+^ | 1.9 | 98.0962 | 397.335 2 | 397.334 5 |
| 73 | 11.53 | C_28_H_49_NO_2_ | 432.384 2 | [M+H]^+^ | 1.4 | 119.088 8  118.087 1 | 431.376 9 | 431.376 3 |
| 74 | 13.60 | C_18_H_34_O_4_ | 313.238 8 | [M-H]^-^ | 1.3 | 119.088 8  118.087 1 | 314.246 1 | 314.245 7 |
| 75 | 14.16 | C_18_H_34_O_4_ | 313.238 9 | [M-H]^-^ | 1.6 | 183.139 4 | 314.246 2 | 314.245 7 |
| 76 | 14.69 | C_18_H_32_O_4_ | 311.223 4 | [M-H]^-^ | 2.0 | 117.929 1 | 312.230 7 | 312.230 1 |
| 77 | 14.75 | C_19_H_38_O_4_ | 353.268 9 | [M+Na]^+^ | 7.7 | 131.086 0 | 330.279 7 | 330.277 0 |
| 78 | 14.79 | C_18_H_30_O_3_ | 295.227 7 | [M+H]^+^ | 3.0 | 119.086 3 | 294.220 4 | 294.219 5 |
|  | 14.83 |  | 293.213 0 | [M-H]^-^ | 2.5 | 117.928 9 | 294.220 2 | 294.219 5 |
| 79 | 14.79 | C_18_H_32_O_4_ | 311.223 4 | [M-H]^-^ | 2.0 | 195.139 4 | 312.230 7 | 312.230 1 |
| 80 | 14.91 | C_18_H_30_O_3_ | 295.227 0 | [M+H]^+^ | 0.7 | 119.086 3 | 294.219 7 | 294.219 5 |
|  | 14.91 |  | 293.213 0 | [M-H]^-^ | 2.5 | 171.1031 2 | 294.220 2 | 294.219 5 |
| 81 | 15.27 | C_18_H_32_O_3_ | 295.227 7 | [M+H]^+^ | 3.0 | 195.139 4  117.929 2 | 294.220 4 | 294.219 5 |
|  | 15.47 |  | 295.228 8 | [M-H]^-^ | 3.1 | 177.055 5  119.050 4 | 296.236 0 | 296.235 1 |








**Figure S1.** The structural formula of compounds identified from F. *unibracteata*, F. *cirrhosa* and F. *thunbergii*.
